# The Thymus/Neocortex Hypothesis of the Brain: A Cell Basis for Recognition and Instruction of Self

**DOI:** 10.3389/fncel.2017.00340

**Published:** 2017-10-27

**Authors:** Silvia Sánchez-Ramón, Florence Faure

**Affiliations:** ^1^Department of Clinical Immunology and IdISSC, Hospital Clínico San Carlos, Madrid, Spain; ^2^Department of Microbiology I, School of Medicine, Complutense University of Madrid, Madrid, Spain; ^3^PSL Research University, INSERM U932, Institut Curie, Paris, France

**Keywords:** cortical neuron diversity, thymus, neocortex, education, self-tolerance, programmed cell death

## Abstract

The recognition of internal and external sources of stimuli, the self from non-self, seems to be an intrinsic property to the adequate functioning of the immune system and the nervous system, both complex network systems that have evolved to safeguard the self biological identity of the organism. The mammalian brain development relies on dynamic and adaptive processes that are now well described. However, the rules dictating this highly constrained developmental process remain elusive. Here we hypothesize that there is a cellular basis for brain selfhood, based on the analogy of the global mechanisms that drive the self/non-self recognition and instruction by the immune system. *In utero* education within the thymus by multi-step selection processes discard overly low and high affinity T-lymphocytes to self stimuli, thus avoiding expendable or autoreactive responses that might lead to harmful autoimmunity. We argue that the self principle is one of the chief determinants of neocortical brain neurogenesis. According to our hypothesis, early-life education on self at the subcortical plate of the neocortex by selection processes might participate in the striking specificity of neuronal repertoire and assure efficiency and self tolerance. Potential implications of this hypothesis in self-reactive neurological pathologies are discussed, particularly involving consciousness-associated pathophysiological conditions, i.e., epilepsy and schizophrenia, for which we coined the term autophrenity.

## Introduction

A basic question of immunology has been the recognition of self from non-self in vertebrates, since the theory proposed by Burnet and Fenner (1949). The self/non-self model, however, does not fully explain how the immune system (IS) works and has been replaced by more fitted theoretical models of immunity that stress the relevance of the context of recognition over the nature of the antigen (Tauber, 2000; Matzinger and Kamala, 2011; Pradeu and Cooper, 2012). Nevertheless, the concept of selfhood remains a valid metaphor to refer to the biological identity of the IS as an autonomous system.

Likewise, the recognition of external and internal sources of stimuli, the outer and the inner world, seems to be an intrinsic property to the adequate functioning of the brain, in what Merleau-Ponty called the “primordial knowledge of the real”^1^ (Merleau-Ponty, 1945). The concept of selfhood for the mind-brain unit has been inherently tied to the concept of self-consciousness and to widespread associative brain connections (Northoff, 2017). While there are diverse philosophical and neurological definitions of consciousness, it is generally conceived as an experienced property of mental activity, which is lost during deep sleep, deep anesthesia and coma (Edelman, 2005). The neural correlate of consciousness is the large-scale cortical wave phase synchronization (Crick and Koch, 1990; Varela et al., 2001) or a multi-stage integration of many microconsciousness (Zeki, 2008). To achieve this emergent property, a stringent cell regulation of the neocortical development contributes significantly to ensure the efficient function of the huge neuronal diversity and tissue homeostasis. It is still unclear to what level of biological organization one can legitimately attribute consciousness to an organism. Thus, understanding the hierarchy of how discrimination of self from non-self takes place at the cellular level is a major challenge, since a cellular basis must underpin high-order functions. If this latter premise is correct, then the basic question is what are the cellular conditions for this self-consciousness.

Several pieces of evidence might suggest that there are common underlying general principles for recognition, memory and learning in the CNS and the IS in vertebrates, even if the mechanisms differ according to each one (Sanchez-Ramon and Faure, 2016). They are both hybrid systems of information’s physics and physiology. In contrast to any other organism’s system, the CNS and the IS share a distinctive multicellular complex network organization, and they are both viewed as cognitive systems, even though at different scales of complexity (Varela and Coutinho, 1991).

Hence, we approached the “logic” driving CNS dynamics with the mindset of an immunologist, by analogy between both evolutionarily adaptive biological systems. T-lymphocyte instruction within the thymus is primarily based on a threshold of affinity to self antigens, yielding an almost limitless repertoire of specific receptors while preventing non-functional and potentially harmful autoimmune reactions. We propose here that during embryogenesis, the discrimination capacity of the nervous self signals would drive the instruction of young neocortical neurons, giving rise to an almost limitless repertoire of specific neocortical neurons. Accordingly, coordinated positive and negative selection processes would take place during cortical neurogenesis as a primary means to avoid non-functional and self hyper-reactive connections, contributing to normal brain functioning and to the primordial knowledge of the real. We shall refer here to the selfhood concept as biological identity in both systems and a prerequisite for self recognition, notwithstanding the obvious differences in complexity of the “CNS self” up to the high-order functions enabling self-awareness, conscience of the consciousness, the mind and the dimension of “I.” We explore herein the hypothesis of a cellular level for the self/non-self discrimination in neurobiology, as a critical assumption based on the prevailing data.

## The Brain’S Thymus Hypothesis

Self discrimination by the IS occurs through complex processes that have been extensively studied but whose mechanisms are not yet fully deciphered (Davis, 2015). The thymus is the central organ of immunologic self/non-self discrimination and of the induction of tolerance (i.e., non responsiveness) to most self antigens, in line with the generation of high T-lymphocyte diversity on grounds of more specific and efficient response. However, this comes at the price of potentially harmful autoimmunity, which would lead to autoimmune disease affecting any cell or tissue, such as multiple sclerosis or diabetes mellitus. Within the thymus, the deletion of highly reactive T lymphocytes to self-antigens and the selection of some self-specific regulatory T lymphocytes (*T*_Reg_) that regulate Ag-specific tolerance (Liu, 2006) constitute the central tolerance. By contrast, the systemic suppression of immune reactivity to self by autoreactive lymphocytes that escaped the “thymic filter” constitutes the peripheral tolerance (Griesemer et al., 2010; Richards et al., 2016). Indeed, T lymphocytes use an evolutionary acquired specific recognition of non-self based on the interaction of T cell receptor (TCR) with exogenous small peptides “presented” in the context of major histocompatibility complex (MHC) molecules. This specific interaction discriminates MHC/“exogenous” peptide from MHC/self peptide complexes within a highly diverse TCR repertoire.

**FIGURE 1:**
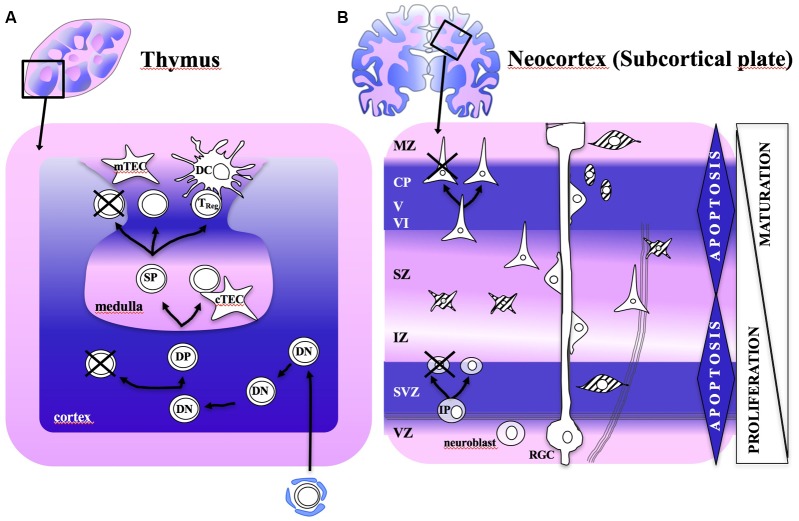
Schematic diagram illustrating the programmed cell death (PCD) waves during development of thymus and neocortex. Tissue is shown according to haematoxylin-eosin staining. **(A)** The thymus is composed by lobes that are distributed into two distinct anatomical areas: the cortex and the medulla. Immature thymocytes derive from bone-marrow precursors, entering the cortex, where positive selection occurs by PCD. Thymic microenvironmental cells within the cortex (cTECs) and the medulla (mTECs and DCs) have an essential role in clonal deletion. Highly promiscuous gene expression pattern by these cells provide the antigen presentation and costimulatory molecules that seem to support negative selection. **(B)** The birth of neurons takes place within proliferative zones adjacent to the ventricles (ventricular and subventricular zones), while postmitotic cells migrate radially to the cortical plate in the surface. GABAergic interneurons migrate tangentially to their final cortical plate destination. PCD affect mostly the proliferating zones (VZ and SVZ), by cell–cell-interaction (positive selection). Postmitotic regions, such as IZ and MZ, where synaptogenesis takes place, are also affected by a second wave of PCD (negative selection). Subcortical plate receives projection neurons from the forebrain, the MTL and the thalamus that are at the core of neocortex architecture and function. Glial cells might also play a relevant role in brain PCD fine-tuning via gap-junction connectivity. cTECs and mTECs: cortex and medullary thymic epithelial cells: DC, dendritic cell; DN, double negative; DP, double positive; IZ, intermediate zone; MZ, marginal zone; SP, single positive; SZ, subplate zone; SVZ, subventricular zone; VZ, ventricular zone.

The intriguing core of thymus instruction of thymocytes toward T lymphocytes relies on the representation of the self by an ubiquitous vast array of self-antigens during embryogenesis and early extrauterine life (Hamazaki et al., 2016), such that immune non-self is understood in terms of all that show intermediate affinity for self (Janeway, 1992). After TCR gene rearrangement and cell lineage commitment, multiple selection processes remove lymphocytes exhibiting too low or too high reactivity for the self by positive and negative selection (**Figure 1**) (Janeway, 1992; Krueger et al., 2016). These two processes can occur sequentially or negative selection can take place directly (Palmer, 2003). Positive selection consists of the selection of T lymphocytes which can interact with self-peptide/MHC complexes, while 90% of lymphocytes which do not appropriately interact with any self-peptide/MHC complex and do not receive the survival signal, are eliminated by programmed cell death (PCD) (Surh and Sprent, 1994). As pointed out above, negative selection refers to the clonal deletion of those TCR-bearing lymphocytes with overly high avidity to self-peptide/MHC (Palmer, 2003). Negative selection is incomplete and some autoreactive T cells exit in periphery (Moon et al., 2011), they are mostly *T*_Reg_ exerting peripheral tolerance (Legoux et al., 2015) to tissue-restricted antigens (i.e., to self). Altogether, only 1% of lymphocytes entering the thymus will become T-lymphocytess (**Table 1**) (Krueger et al., 2016). The whole process was initially conceptualized by Ehrlich (1902) as the “horror autotoxicus,” which in his own words “prevents the production within the organism of amboceptors directed against its own tissues,” as otherwise it would be “dysteleological in the highest degree”.

**Table 1:** Comparative scheme showing key parameters for education on self according to the proposed model.

Education on self	Immune system	Nervous system
WHO	T-lymphocyte	Neocortical neuron
WHEN	Embryogenesis and early life	Embryogenesis and early life
WHERE	Thymus	Subcortical plate of neocortex
HOW	POSITIVE SELECTION: elimination of T-lymphocytes whose TCRs fail to engage MHC-peptide and do not receive survival signal. PCD ≈90% of immature thymocytes. Takes place in the cortex. NEGATIVE SELECTION: elimination of T-lymphocytes whose TCRs engage the peptide-MHC with high affinity (self-reactive T-lymphocytes). PCD ≈50% of mature lymphocytes. Mainly in the medulla, can occur in the cortex.	POSITIVE SELECTION: elimination of neurons that do not succeed in establishing adequate connections with close cells. PCD ≈80% of progenitor neurons. Takes place in the VZ and SVZ. NEGATIVE SELECTION: elimination of neurons that show excessive reactivity to self signals (self-reactive neuron). PCD ≈50% of mature neurons. Mainly in the IZ, SP, and MZ.

*PCD, programmed cell death; TCR, T-cell receptor; IZ, intermediate zone; SP, subplate zone; MZ, marginal zone*.

The evolutionary neocortical expansion in mammals’ brain, and more particularly in humans, is thought to provide a basis for their remarkable cognitive abilities (Wilsch-Bräuninger et al., 2016). This neocortical expansion together with concomitant neuronal diversity accounts for the extreme precision of recognition and explicit memory of the sensations or *qualia* and of motor skills that may enable the possibility of self-consciousness, up to the degree of fineness distinctive of humans. Each major sensory and motor modality has a corresponding area in the neocortex. Self-produced cues could be defined as the signals arising within the body, in contrast to non-self exteroceptive signals, including those generated as an actor. Examples of non-self cues are the direct perception of an external voice or image; the normal (tolerant) self cues are the recall of a voice or an image that are perceived as internal in origin. By contrast, their hyper-reactive self cues counterparts are hallucinatory experiences of internal voice or visual image that are internal in origin but perceived as external reality. In our hypothesis, we are thus left in the cellular general mechanisms underlying self-recognition (biological self) by both systems. One of the principles underlying neocortical expansion has been the increase in neuronal generation, mainly at the expense of highly proliferative basal progenitors typically in the subventricular zone (SVZ) (Lui et al., 2011). As mentioned above, this immune-based analogy does not pretend to explain emergent brain processes accounting for intelligence, the complex human behavior and the set of properties that we call mind (mental self), which are currently not completely covered by neurobiology, but beyond, by psychology and sociology. To state the problem in other words, even if we reach a complete knowledge on the neurological basis of consciousness, this does not imply that we have a complete knowledge of the nature of consciousness (Gabriel, 2015), or that we know more than Shakespeare about human nature.

Turning to selfhood and the brain, we hypothesize that the nervous system evolved to discriminate perceptible non-self from non-perceptible self. It would imply that neocortical neurons were instructed during the neurogenesis by recognition of the vast array of self-produced cues. The subcortical plate (**Figure 1**) would correspond to the brain thymus. Until we know more about the specific requirements for such recognition, we could speculate that those neurons capable of interacting with self cues at intermediate range would be positively selected, while those both with too weak or high interaction for self-signals would be removed. As a result, a minimum proportion of functional neurons (estimated around 10% in humans, with limited experimental data) (Gohlke et al., 2007) survive to recognize a limitless array of signals to confront the perceptible world (**Table 1**). We briefly outline the main arguments supporting the advanced self-criteria selection that are shared with the IS, as follows:

(1) The self principle. Both the IS and the CNS are identity-centered systems. Similarly to the IS, in which the self/non-self criterion governs the functioning at the cell level, we favor the notion of self/non-self as a primary criterion governing the functioning at the cell level within the cerebral neocortex.
(2) The need for self-tolerance. The evolution of the adaptive IS with a highly diverse TCR repertoire that enables a more specific and efficient response comes at the price of potentially harmful autoimmunity. As mentioned above, thymic selection is the bastion of T lymphocyte diversity and specific central tolerance. Similarly, evolutionary expansion in cortical size and folding and the laminar differentiation of the neocortex comes along with an extremely high diversity and specialization of new neurons that account for the exponential development of intelligence in the mammal brain (Northcutt and Kaas, 1995). As an evolutionary imperative, it also exacts a high price of the hyper-recognition of self signals that would interfere with brain functioning. To solve the self-recognition problem, similar common principles to the IS and the CNS may underlie the preservation of high specificity together with tolerance to self.
(3) *In utero* education processes. The self/non-self discrimination machinery is mostly attained during intrauterine development and early life, with progressive involution of the thymus at postnatal life (Hamazaki et al., 2016). Similarly, neurogenesis of neocortical neurons is mainly restricted to embryogenesis and early postnatal life (Bhardwaj et al., 2006), with large differences between humans and other mammals in the output of new neurons from the subcortical plate (Ming and Song, 2011). In both the IS and the CNS, PCD is tightly regulated by pro- and anti-apoptotic genes serving specific developmental functions (Yeo and Gautier, 2003; Zhang et al., 2005).
(4) The self-test. A coordinated multiple-step selection processes take place within the thymus in a specific spatiotemporal manner during development to assure the elimination of useless and highly self-reactive T lymphocytes mainly by positive and negative selection. Likewise to what happens in the thymus, an initial overproduction of cells in the vertebrate CNS will have to be adjusted to functional needs (Cowan et al., 1984). Massive and widespread rates of neuronal PCD are much more prevalent in vertebrates during corticogenesis (Cowan et al., 1984), with respect to invertebrates (Buss et al., 2006). We further evaluate below the concept of self-test in the context of experimental results of neocortical neurogenesis.

## Attempt for Biological Characterization of the Self-Test

Current hypotheses of corticogenesis do not explain the principle governing the processes that give rise to the extreme precision and complexity of developing neural circuits, leaving room for random events’ interpretation (Dekkers and Barde, 2013; Mosley et al., 2017). The pioneering neurotrophic hypothesis (Oppenheim, 1989) stated that those neurons establishing effective and functional connections better compete for neurotrophic factors and are more likely to survive (Buss et al., 2006; Stiles and Jernigan, 2010). Currently, this theory mainly applies for peripheral nervous system (Mosley et al., 2017). Neuronal death, according to the revised “systems matching” hypothesis, is envisioned both as a mechanism for correcting errors in neuronal production, migration or branching and ensuring distal connectivity (Buss and Oppenheim, 2004; Hyman and Yuan, 2012; Chen et al., 2013; Dekkers et al., 2013) or to get rid of cells that have accomplished a transient function in brain development (de la Rosa and de Pablo, 2000).

It would be reasonable to suppose a selection principle to build up the striking specificity in neurons’ geometry, location and function. We hypothesize that the vast range of self-induced electrical activity represented during neurogenesis would drive selection and migration of neocortical neurons for adequate performance in recognition and memory during postnatal life. The mechanisms accountable for the neocortical neuron repertoire remain unknown, recent findings suggesting a unique somatic mutation fingerprint in each cortical neuron (Evrony et al., 2012). Similarly to thymic selection, a dual excitability threshold for self electrical cues would avoid too weak excitability putatively responsible for non-functional connections or excessively high excitability putatively responsible for self-reactive responses.

We present herein experimental findings on neurogenesis that could be predicted by our self/non-self hypothesis. Compatible with this line, a remarkable 2-wave neuronal loss has been described during corticogenesis. The major wave affects cell proliferation zones (ventricular and subventricular zones) before establishing synaptic connections. The second wave, less extensive than the first one, affects postmitotic zones (intermediate zone, cortical plate, and marginal zone), where synaptic connections are being formed (Blaschke et al., 1996; Buss et al., 2006; Mosley et al., 2017) (**Figure 1B**). The two PCD waves are regulated by distinct proapoptotic genes (Kuan et al., 2000). Therefore, PCD seems to have a major role in the selection of appropriate neurons aimed to CNS homeostasis during development, and elimination of expendable or potentially harmful cells. We assign the first PCD wave to positive selection, when only a minority of cells is active, mostly as sporadic calcium spikes corresponding to immature action potentials (Allene et al., 2008; Allene and Cossart, 2010); whereas we assign the second wave to negative selection, when matching between neurons and their targets in the postmitotic regions occurs, as follows:

Positive selection of self-responsive neurons: In the murine cerebral cortex, dying cells are rare at early stages (embryonic day, E10, <1%), but reach a peak of 70% at E14 and 50% at E18. Although no direct experimental evidence exists relating PCD to self recognition, prominent spontaneous voltage-dependent activity and intracellular calcium transients (E16) (Allene et al., 2008) may modulate PCD, as active neurons will evolve to synchronized small networks at this phase (Luhmann et al., 2016). This early-phase PCD was interpreted as a means to regulate the size of the precursor population for the selection of adequate neuronal sublineage clones, according to genetic and phenotypic studies (Blaschke et al., 1996; Yeo and Gautier, 2003; Buss et al., 2006). In our view, a screening of excitability may be borne by appropriate cell–cell interactions with adjacent cells as a means of communication fitness, by which those proliferating cells that are not able to interact adequately, or expendable neurons, will undergo PCD, thus as a positive selection mechanism.

Negative selection for highly self-reactive neurons: Recent evidence indicate that genetic and epigenetic mechanisms, and the involvement of electrical activity account for the early cerebral cortex construction (Spitzer, 2006). The resulting coherent neuronal activity patterns suggest a remarkable fine-tuned spatiotemporal regulation to adequately shape synapse-driven neural circuits. Supporting a synapse-driven selection of cortical neurons is the fact that this second PCD wave takes place once the axon has already reached the target field (Cowan et al., 1984). For instance, a dramatic PCD peak of cortical neurons occurs at E18 within the murine cingulate cortex area 2, after some of them have crossed the midline and established connections with the opposite hemisphere and the hippocampus (Mosley et al., 2017). On the other hand, adhesion G-protein-coupled receptors (GPCR) seem to link synapsis formation and PCD (Langenhan et al., 2016). Expression of excitatory and inhibitory neurotransmitters modulate PCD and neural plasticity (Kanold and Luhmann, 2010). Indeed, sequential waves of specific neurotransmitters, i.e., glutamatergic- followed by GABA-driven synapses, seem to be shaping coherent neural patterns in the rodent developing neocortex shortly after birth (Allene et al., 2008; Allene and Cossart, 2010).

Neocortical neuronal connections seem to be guided by early (E13) afferent projections at the intermediate zone from the basal forebrain (Koh and Loy, 1989) and by thalamocortical circuits (Kanold and Luhmann, 2010). All those afferences stress the relevant role played by more ancient structures in shaping neocortex’ PCD, architecture and function (Sanchez-Ramon and Faure, 2016). In this setting, the characteristic subplate location of these afferences in mammals represents a key factor favoring neocortex expansion, laminar differentiation of the cortical plate and neuronal diversity (Super and Uylings, 2001). Altogether, this second PCD wave, occurring after neural differentiation, would serve to fine-tuning connectivity and to avoid excessive reactivity to self signals. PCD is extended after birth. At birth, gap-junction coupling, neurotransmitter receptors and synapses-driven transmission will result in the emergence of coherent activity patterns of neural networks during development (Somogyi et al., 1998; Allene et al., 2008, 2015).

Inhibitory or suppressive cells for central tolerance: Similarly to the immunomodulatory function of *T*_Reg_ output from the thymus, there is an excitatory/inhibitory switch of γ-aminobutyric acid-(GABA)ergic neurons on the cortical neural network that shapes the vertebrate neocortex (Tyzio et al., 2007; Kirmse et al., 2015; Valeeva et al., 2016). This switch seems to be specific of neuronal type and of developmental stage (Luhmann et al., 2014). Here also the projections from thalamus seem to have a key role in the maturation of cortical inhibition (Allene et al., 2008).

## Anti-Self Pathology: Autoimmunity and Autophrenity

The clinical expression of altered self/non-self immune discrimination turns out in an imbalance between immunodeficiency and autoimmunity. Autoimmune diseases are attributed to the interplay of multifactorial intrinsic (genetic, immunoregulatory, endocrine, and neuropsychological), extrinsic factors and yet stochastic events that converge from latent status to clinical relapse that can be immunomodulated (Fernández-Paredes et al., 2016). Translated to pathologies of the CNS, although the nosology has not been addressed in terms of self-recognition, we might also identify a spectrum between mental retardation and hyper-reactivity to self. For the latter and in parallel to autoimmunity, we coined the term **autophrenity** (*et. gr.*φρ*iv* phrēn, mind, brain). In parallel with autoimmunity, autophrenity would result from multifactorial intrinsic (genetic, neurorregulatory, endocrinological, psychological, and immunological), extrinsic and stochastic factors conveying from latent stages to clinical onset of the disease. We will examine two examples of autophrenity, schizophrenia and epilepsy, which are associated with altered self-consciousness.

Schizophrenia arises from the interplay of genetic, immunologic, endocrine, neuropathological, psychodevelopmental and socio-environmental factors. Schizophrenia is a severe mental disorder characterized by alteration of patient’s functionality and activity, with anomalous self-experiences, such as auditory hallucinations, hearing of voices, delusions, avolition of emotional expression and diverse cognitive disturbances. Genetic susceptibility and neurodevelopmental alterations that remain in a latent status until immunological, environmental factors and maturational changes trigger clinical onset has been proposed (Insel, 2010). During cortical neurogenesis in schizophrenic patients, one of the most consistent findings is the deficit of inhibitory interneurons, mainly in GABAergic inhibition, inducing overstimulation of dopaminergic neurons (Catts et al., 2013). Other neurotransmitters systems are disrupted, although with controversial results in the complex networks of excitatory glutamatergic (particularly, NMDA hypofunction) and modulating dopaminergic receptors (Chinchilla, 2007). GPCR mutations’ association with schizophrenia suggests altered neurogenesis and resultant excitability in schizophrenia pathophysiology (Devor et al., 2017). There is no cure for the disease, and classical treatment strategies have been directed to block dopamine D2 receptors, with limited results. We could speculate that the predominant inhibitory defect induces an imbalance toward excitatory activity with activation of self-reactive cortical pyramidal neurons that eventually declare at disease onset, banishing the boundaries from perception, with the known devastating consequences for the life of the patients.

Epilepsy encompasses a group of disorders characterized by paroxysmal and excessive electrical neuronal discharges at any region of the cerebral cortex. Besides interfering with the normal functioning of the affected region, impaired or lost overall level of consciousness is common, but even focal seizures associate selective deficits in consciousness and thus self consciousness (somatosensory symptoms, visual or auditory delusions, illusions, vertiginous or complex hallucinations, etc.) (Blumenfeld, 2012; Blumenfeld and Meador, 2014). Our current understanding of the molecular basis of epilepsy has been challenged by the study of rare hereditary forms. The genetic defect (monogenic or combinatorial) involves in virtually all types channels/receptors and neurotransmission processes affecting specific neocortical regions with enhanced excitatory over inhibitory synaptic connectivity (Symonds et al., 2017). Besides, neurogenesis, disturbances in PCD or neuronal migration disorders, resulting in focal cortical dysplasia, have been recently established to be the most common cause of long-term hyperexcitability and epileptic seizures in children and adolescents (Palmini and Holthausen, 2013; Luhmann et al., 2014). Here again, mutations in GPCR are associated with wide range of seizures and cognitive deficits, suggesting their role on neuronal selection (Symonds et al., 2017). Focal cortical dysplasia is associated with severe epilepsy, disclosing the combination of excess of dysmorphic or bursting neurons and abnormal distribution of inhibitory neurons at epileptogenic areas (Palmini et al., 1991; Ferrer et al., 1992; Castano de la Mota et al., 2017). Hyperexcitability of dysplastic foci has raised the concept of intrinsic epileptogenicity (Palmini, 2010), which could correspond in our view to the predominance of highly self-reactive neurons that unbalance neural homeostasis. A defect in GABAergic inhibition has been described in experimental models of epilepsy (Zhu and Roper, 2000; Cossart et al., 2001). This fact, together with the modulation of MTL (Yu et al., 2017) and thalamocortical connections suggest the role of unbalanced excitatory over inhibitory neurotransmission breaking self tolerance in the genetically predisposed individual with acquired epilepsy.

The etiological propositions for these illustrative disorders led alternatively to the conclusion that the self principle may be a new and unexpected element entering in CNS pathology.

## Discussion and Conclusion

We have developed a mechanistic hypothesis of self recognition in the brain at the cell level from an immunological perspective. The theoretical development of this hypothesis has led us to the proposition of a thymus-like model of cortical neurogenesis based on this scenario. The aim of our work is justified, by one hand, because the central question of a selfhood brain cell basis has been surprisingly neglected, mostly due to the assumption that consciousness (and thus self-consciousness) is the result of broad integrated connections among all parts (both as neural correlates of a conscious perception or as a “global neuronal workspace,” see Dehaene and Changeux, 2011; Tononi et al., 2016); but also because it was approached from a main physics/mathematical perspective. Some of the most important attempts of scientifically addressing the issue of self neural cell basis, from Leibniz, who claimed the logic of a cell awareness or *apperception* (Leibniz, 2017), to Penrose, who explains cell conscious experience based in quantum physics (Penrose, 1989). Hameroff and Penrose (2014) hypothesis proposes that microtubules of neuron cytoskeleton accounts for discrete events of orchestrated objective reduction status corresponding to moments of consciousness. On the other hand, it is justified also because there is to date no satisfactory explanation of the mechanisms behind the neocortical neurogenesis driving the almost limitless specificity of the neuronal repertoire, which settle the basis of self/non-self discrimination. To obtain an answer to the question of a self cell brain basis, we argue that we have to ask how the immune system has resolved it, because it is biology (and then coupled with chemistry and physics) that can better explain diversity and specificity through evolution. To the best of our knowledge, this hypothesis has not been previously posed from this standpoint.

There are compelling experimental evidence and clinical observations that might support the contention that a cellular basis of brain selfhood exists and drives cortical neurogenesis. The neocortex shows an extraordinary topographical distribution of sensory and motor areas, in which a complex interplay of specific excitatory and inhibitory (E/I) neurons seems to ensure efficiency and self tolerance. The relevance of this E/I balance for the self/non-self discrimination is evidenced by that impairments in the E/I balance in a particular modality neocortex may elicit hyper-excitability to self cues, which in turn may render the individual susceptible to abnormally excessive perception or motor responses and compromises consciousness. This phenomenon has been typically described for epileptic seizures (Fisher et al., 2005; Sakakibara et al., 2012; Hirose, 2014; Wimmer et al., 2015). Moreover, disturbed E/I balance has been associated with several neuropsychological disorders, such as schizophrenia, autism and others (Zhang and Sun, 2011), which could thus be considered as anti-self brain pathologies (autophrenity). Under our hypothesis, the E/I balance would result from tightly coordinated processes of selection during neurogenesis that warrant self/non-self discrimination and avoid hyper-excitability against self cues. Compatible with this, abnormal cortical neurogenesis with deficient neuronal apoptosis and migration results in intellectual disability of diverse degree (agyria, pachygyria) and epilepsy (Fitzgerald et al., 2011). For instance, recent mutations in CRADD (caspase-2-mediated neuronal apoptosis) results in severe pachygyria and epilepsy (Di Donato et al., 2016). By contrast, abnormally high rate of apoptosis is generally associated with neurodegenerative diseases (Down syndrome, Alzheimer disease, Huntington disease, dementia) (Busciglio and Yankner, 1995; Arendt, 2001; Hyman and Yuan, 2012). In addition, dysregulation of neocortical neurogenesis is at the basis of schizophrenia and other psychotic disorders, although with more complex multifactorial etiology that mark its clinical onset. On the other hand, neuromodulation seems to be a plausible therapeutical strategy for suppressing excessive anti-self hyper-excitability, as transplantation of inhibitory interneurons reduces focal ictal activity (Casalia et al., 2017). Indeed, a diverse array of inhibitory neurons actively modulate excitatory activity in a context-dependent manner and shape the excitatory circuits during experience-dependent plasticity and learning (Hattori et al., 2017). Finally, with respect to potential self-selection processes by neural host tissues, an *in vitro* model of hyper-excitable epileptic tissue grafted with pluripotent stem cell neural progenitors (PSC) showed selective host-to-graft synaptic afferents from hippocampus and a modulation toward GABAergic differentiation (Avaliani et al., 2014). *In vivo* evidence of the host modulation of cortical neurons derived from human induced PSC has been shown for the first time in an adult stroke-injured rat model (Tornero et al., 2017). The authors demonstrate that only some of the afferent inputs from the host into selected grafted cells become functional and are integrated in the host circuitry. As they point out “the magnitude of efferents and afferents and level of functional integration after transplantation of mouse embryonic SC-derived neurons into adult mouse brain are mainly dependent on the degree of identity match between transplanted cells and injured cortex.” Again, self-guided selection processes stemming from host nervous system into grafted PSC are suggested.

With our brain’s thymus hypothesis, we seek to probe a more comprehensive explanation on the founding principles for neocortical development in terms of self/non-self recognition. From this self principle, pathogenic processes of several neurological diseases and therapeutical strategies could be reappraised and could raise the possibility of unexpected feedbacks and applications in an open interdisciplinary context. Conceivably, the self principle could also open a new understanding of the cellular basis of self-consciousness and the own working of the CNS. We hope this hypothesis could fertilize the debate on the scope of developmental neurobiology and that the self principle could be integrated in the further studies required to decipher the neuronal selection.

## Author Contributions

SS-R and FF have equally contributed to the conception and design of the manuscript, drafting, critical revision and final approval of the article. SS-R and FF agree to be accountable for all aspects of the work in ensuring that questions related to the accuracy or integrity of any part of the work are appropriately investigated and resolved.

## Conflict of Interest Statement

The authors declare that the research was conducted in the absence of any commercial or financial relationships that could be construed as a potential conflict of interest. The reviewer PP and handling editor declared their shared affiliation.

## Footnotes

1 “Car si je peux parler de «rees» et de «réalité», m’interroger sur la distinction de l’imaginaire et du réel, et mettre en doute le «réel», c’est que cette distinction est déjà faite par moi avant l’analise, c’est que j’ai une expérience du réel comme de l’imaginaire, et le probléme est […] d’expliciter notre savoir primordial du «réel», de décrire la perception du monde comme ce qui fonde pour toujours notre idée de la vérité.” *Op. cit.* p. 22.
